# The unfolded protein response machinery in glioblastoma genesis, chemoresistance and as a druggable target

**DOI:** 10.1111/cns.14839

**Published:** 2024-07-17

**Authors:** Lucette Z. Simbilyabo, Liting Yang, Jie Wen, Zhixiong Liu

**Affiliations:** ^1^ Department of Neurosurgery, Xiangya Hospital Central South University Changsha Hunan China; ^2^ Hypothalamic Pituitary Research Center, Xiangya Hospital Central South University Changsha Hunan China; ^3^ National Clinical Research Center for Geriatric Disorders, Xiangya Hospital Central South University Changsha Hunan China

**Keywords:** chemoresistance, glioblastoma, glioblastoma resistance, glioblastoma stem cell, temozolomide, unfolded protein response

## Abstract

**Background:**

The role of the unfolded protein response (UPR) has been progressively unveiled over the last decade and several studies have investigated its implication in glioblastoma (GB) development. The UPR restores cellular homeostasis by triggering the folding and clearance of accumulated misfolded proteins in the ER consecutive to endoplasmic reticulum stress. In case it is overwhelmed, it induces apoptotic cell death. Thus, holding a critical role in cell fate decisions.

**Methods:**

This article, reviews how the UPR is implicated in cell homeostasis maintenance, then surveils the evidence supporting the UPR involvement in GB genesis, progression, angiogenesis, GB stem cell biology, tumor microenvironment modulation, extracellular matrix remodeling, cell fate decision, invasiveness, and grading. Next, it concurs the evidence showing how the UPR mediates GB chemoresistance‐related mechanisms.

**Results:**

The UPR stress sensors IRE1, PERK, and ATF6 with their regulator GRP78 are upregulated in GB compared to lower grade gliomas and normal brain tissue. They are activated in response to oncogenes and are implicated at different stages of GB progression, from its genesis to chemoresistance and relapse. The UPR arms can be effectors of apoptosis as mediators or targets.

**Conclusion:**

Recent research has established the role of the UPR in GB pathophysiology and chemoresistance. Targeting its different sensors have shown promising in overcoming GB chomo‐ and radioresistance and inducing apoptosis.

## Graphical abstract

The Unfolded protein response is suggested as a therapeutic target for glioblastoma. The unfolded protein response is activated in response to endoplasmic reticulum stress in glioblastoma cells. Its three arms IRE1, PERK, and ATF6 activate their downstream pathways furthering tumor cell survival, proliferation and the development of chemoresistance. Furthermore, targeting the UPR arms induces apoptosis in glioblastomacells. Adapted from "UPR signaling (ATF6, PERK, IRE1)", by BioRender.com(2024). Retrieved from https://app.biorender.com/biorender‐templates.

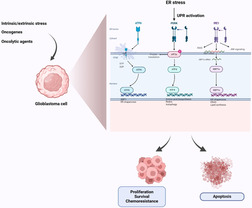

## 1 INTRODUCTION

Tumor cells go through both oncogenic and environmental stresses during different phases of their development. A consecutive increase in protein secretion due to the high nutrient demand is observed, leading to the accumulation of improperly folded proteins in the endoplasmic reticulum (ER). This situation of the accumulation of misfolded proteins in the ER is called ER stress. It triggers the activation of a pro‐survival adaptative mechanism termed as the “unfolded protein response” (UPR); an intrinsic adaptative mechanism that regulates proteins homeostasis and salvages the cell.^1^ In fact, the UPR activation aims at reducing the load of misfolded proteins via their refolding, clearance or autophagy, and in case it is overwhelmed it induces apoptosis.

Over the past 15 years, the role of the UPR in tumor biology has been progressively unveiled. The UPR has three membrane sensors that are activated in response to oncogenes and overexpressed in Glioblastoma (GB).^2^ It modulates different stages and processes of GB progression from GB stem‐like cells (GSCs) development, cell transformation, unrestricted proliferation, angiogenesis, tumor microenvironment (TME) to invasion.^3^, ^4^ Moreover, the UPR is associated with GB malignancy, chemoresistance, recurrence, and a worse prognosis. Hence, several studies on how the manipulation of the UPR sensors could alter GB progression, resistance to treatment, and relapse have been conducted.

Herein, we survey the implication of the UPR in the cell physiology, tumorigenesis, then in GB progression, and in the development of treatment resistance. Furthermore, we review and discuss the current avenues and prospects for treatment through the identification of targeted therapies.

## 2 PHYSIOLOGY OF THE UNFOLDED PROTEIN RESPONSE

The ER is the first machinery of the secretory pathway which is in charge of the synthesis of a substantial fraction of proteins, their modification, assembly, and delivery to specific destinations in eucaryotic cells.^5^ Intrinsic and extrinsic cellular stressors such as nutrient deprivation, depletion of calcium, pH alteration, somatic mutations, sustained demand on the secretory pathway, to name a few, trigger the accumulation of unfolded or misfolded proteins in the ER.^6^ The subsequent buildup of proteins leads to a dysfunction of the folding machinery in the ER causing ER stress, which threatens the cell viability. Hence, to survive and thrive, the cell will activate different cellular‐stress response pathways among which the UPR. The UPR is an adaptative cellular response made of a series of signal transduction cascades aiming inter alia at relieving the ER stress. The UPR restores proteostasis by increasing ER chaperone transcription and limiting protein secretion.^7^ Furthermore, it increases the degradation capacity to allow for more protein clearance. Thus, salvaging the cell from the deleterious stress. However, when overwhelmed, it induces apoptotic cell death. The UPR has three ER‐anchored transmembrane stress sensors, inositol requiring enzyme 1 (IRE1), protein kinase RNA‐activated‐like ER kinase (PERK), and activating transcription factor 6 (ATF6). They are activated in response to ER stress and maintained inactive in a normal state by their association with 78 kDa glucose‐regulated protein (GRP78) also called binding immunoglobulin protein (BiP).^8^, ^9^ Under ER stress, the three sensors dissociate from GRP78 for their oligomerization, autophosphorylation, and the activation of their respective downstream pathways.^10^

When the UPR is activated, PERK phosphorylates the α‐subunit of eukaryotic initiation factor 2 (eIF2α) resulting in a selective inhibition of protein translation. With eIF2α phosphorylation, activating transcription factor 4 (ATF4) translation is upregulated, which promotes cell survival by targeting the translation of genes and factors involved in cell survival along with those involved in cell apoptosis, such as the transcription factor C/EBP homologous protein (CHOP).^10^ In addition, ER oxidoreduction is activated by CHOP inducing apoptosis through the release of calcium in the cytoplasm from the ER.^11^ PERK has an immediate response to stress and a strong induction of CHOP when the UPR is overwhelmed. Hence, PERK is mainly pro‐apoptotic. However, the factors and balance governing the overexpression of ATF4 for survival and that of CHOP for apoptosis are not clearly understood.

When the UPR is activated, IRE1 autophosphorylates and activates its cytoplasmic nuclease domain, which cleaves the X‐box binding protein 1 (XBP1) to generate its active form XBP1s by splicing XBP1 mRNA, inducing the upregulation of cytoprotective genes, chaperones, and other UPR target genes.^8^, ^12^ In addition, the anti‐apoptotic Bcl‐2 proteins, can indirectly modulate IRE1 by enhancing XBP1.^13^ On the other hand, IRE1 can trigger a pro‐death signaling by binding to the tumor necrosis factor receptor‐associated factor 2 (TRAF2) and inducing c‐Jun N‐terminal kinase (JNK) activation. IRE1 is the most regulated of the three UPR sensors^10^ and considered primarily pro‐survival. IRE1 and PERK phosphorylation are critical regulatory mechanisms for UPR activation.^12^ They are the most studied UPR sensors.

When the UPR is activated, ATF6 is transported into the Golgi apparatus, cleaved, and releases a basic leucine zipper transcription factor (ATF6f), that triggers gene expression of UPR response units such as ER chaperones, XBP1, and CHOP.^14^, ^15^ XBP1 connects ATF6 to pro‐survival IRE1‐emitted signals.^10^ ATF6 is deemed primarily pro‐survival and is the less studied of the three sensors.

The UPR is closely associated with autophagy, a catabolic process that degrades damaged organelles, large protein clusters, misfolded and dysfunctional proteins in autophagosomes to maintain cellular homeostasis.

The UPR regulates several embryologic and adult stem cell biological processes and the regulations are cell‐type specific. For example, the UPR sensors modulate direct and indirect neurogenesis, neuron self‐renewal, differentiation, and viability and have a role in cell fate acquisition throughout mammalian brain development.^16^, ^17^, ^18^, ^19^, ^20^, ^21^ Moreover, the UPR is associated with the viability of neuronal stem cells (NSCs), which are found in the subventricular zone (SVZ) and subgranular zone (SGZ) of the hippocampal dentate gyrus.^22^, ^23^ GSCs are thought to be derived from the subventricular NSCs^24^, ^25^ and the UPR is implicated in the viability of adult NSCs in the SVZ.

Abnormal UPR signaling and disrupted ER proteostasis are associated with several pathologies such as chronic inflammation,^26^ neurodegenerative^27^, ^28^ and metabolic diseases,^29^ and different cancers including GB.

Overall, despite the fact that the balance between the cytoprotective and apoptotic functions of the UPR and how the fate of cells is decided is yet to be fully understood, its function in cell homeostasis has been progressively established as reported by the findings above. The UPR is a quality control pathway with two opposite cellular outputs in response to ER stress. In the effort of homeostasis maintenance, the UPR decreases the load of unfolded proteins and slows down the secretory pathway. In contrast, when overwhelmed, it triggers cell death by apoptosis. In other words, depending on factors such as the type of sensor activated and the type and intensity of the response, the UPR promote cell survival or induce apoptotic cell death. It is integral to cellular homeostasis and survival.

Beyond its role in cell physiology, the UPR is associated with tumor biology. How the UPR is involved in tumor cell development and survival is discussed below.

## 3 THE UNFOLDED PROTEIN RESPONSE IN TUMOR DEVELOPMENT

Recent discoveries have unveiled the implications of the UPR at different stages of tumor development.

### 3.1 Tumor initiation

**FIGURE 1:**
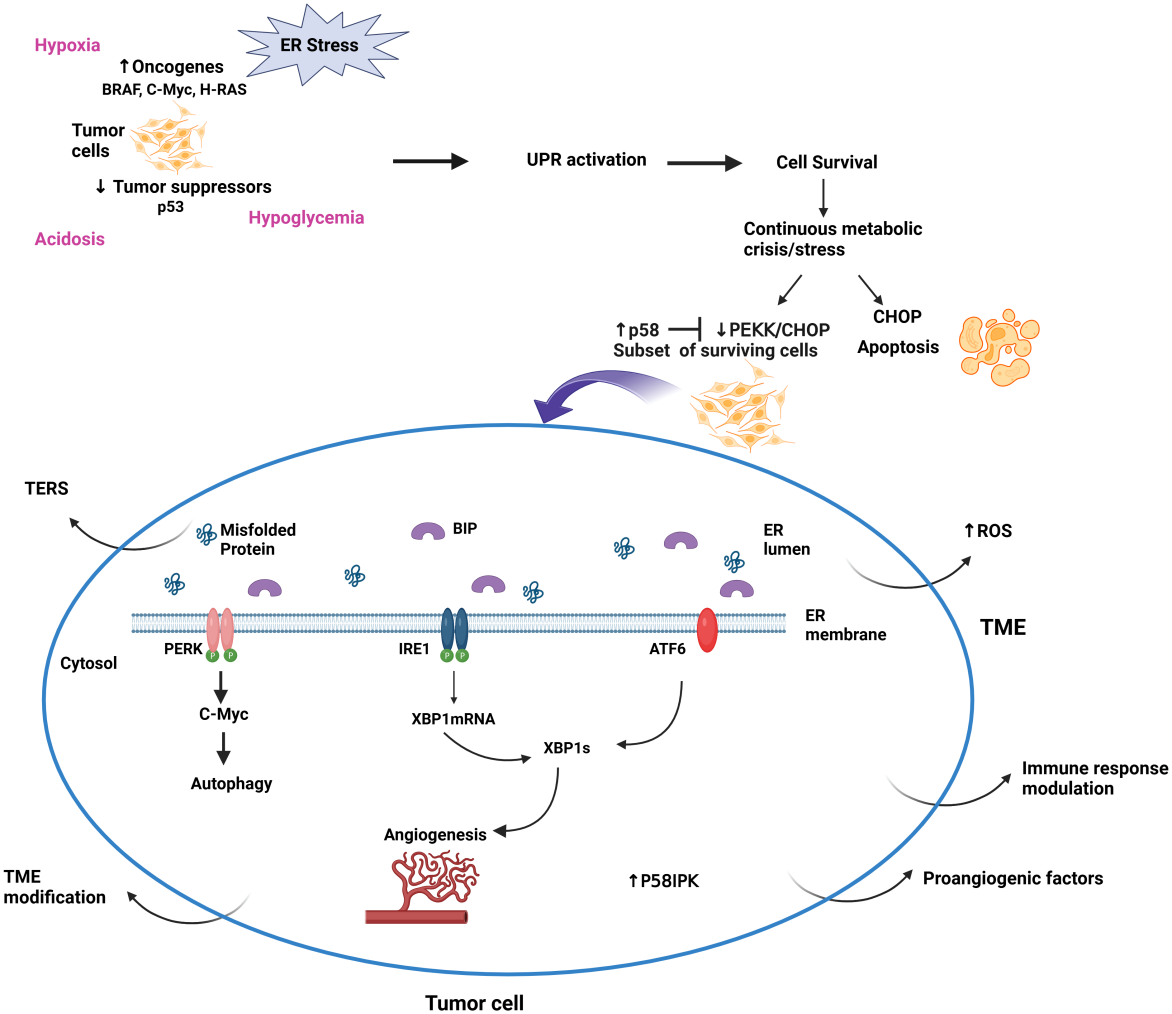
The unfolded protein response: implication in tumor. The “permanent” stress to which tumor cells are submitted triggers the activation of UPR which promote cell survival or apoptosis when the overload of protein cannot be cleared. The surviving cells will have high p58 with an inhibition of CHOP. The UPR modulate angiogenesis, autophagy and the TME via its modification, immune response modulation, secretion of proangiogenic factors, TERS and increase in ROS. Created with BioRender.com

The upregulation of oncogenes such as BRAF^V600E^, c‐MYC, and H‐RAS or the downregulation of tumor suppressors such as p53 are at the core of tumor initiation. They induce cellular transformation with subsequent uncontrolled and rapid cell division, overwhelming the ER and its folding capacity. The three branches of the UPR: PERK and IRE1 are activated in response to oncogenes and ATF6‐dependent pathways are activated to a lesser extent to promote the tumor initiation.^30^ In fact, the three branches activate their downstream pathways to correct the load of unfolded /misfolded proteins and meet the increased protein folding and secretory demand. Moreover, PERK/eIF2α/ATF4 signaling mediates autophagy and protein degradation in response to the proto‐oncogene c‐Myc to promote cell survival^7^, ^31^ (Figure 1).

### 3.2 Intrinsic and extrinsic stress

As mentioned earlier, tumor cells are subjected to several intrinsic and extrinsic perturbing factors that induce selection pressure. The extrinsic factors or restrictive conditions in the TME to which tumor cells are exposed include hypoxia, acidosis, nutrient deprivation, or chemotherapies. In addition, solid tumors are exposed to different intrinsic factors such as oncogenic activation, exacerbation of secretory capacity, and chromosome number disruption, responsible for the high demand for protein.^32^ The preceding will lead to the accumulation of unfolded and misfolded proteins which in turn will trigger the activation of the UPR in an initial attempt of the tumor cell to survive. The activated UPR will promote cell survival and transformation and eventually the development of different tumor features essential to their progression.

### 3.3 Metabolic crisis and neoangiogenesis

Hypoxia, limited glucose availability, and acidosis characterize the harsh environment at the early stage of tumor development resulting from the rapid cellular proliferation and growth and the use of glucose as principal fuel. In fact, the rapid proliferation of tumor cells outgrows their blood supply leading to heterogeneous oxygen availability within the tumor with elevated ROS. The resulting metabolic crisis triggers the ER stress and UPR signaling leading to ER stress‐induced cell death with elevated CHOP. A subset of surviving cells to the metabolic crisis, tumor‐initiating cells, will acquire an adaptative pattern with increased p58 expression and suppress PERK‐CHOP signaling.^33^ P58 is an inhibitor of PERK,^34^ a co‐chaperone,^35^ and promotor of GRP78 activity.^36^ In addition, there is a crosstalk between p58 and ATF6 to enhance cell survival at this stage.^37^, ^38^ Consequently, the surviving cells will activate the cytoprotective mechanism with p58IPK and switch the UPR to its pro‐survival side. In addition, IRE1 and PERK are associated with tumor cells thriving in hypoxic and hypoglycemic environments.^39^, ^40^, ^41^ The UPR has a role in the secretion of pro‐angiogenetic factors through IRE1‐XBP1 or via the indirect regulation of XBP1mRNA by ATF6.^30^, ^42^ In fact, the deficiency in the protein disulfide oxidase ERO1 alpha blunted the expression of hypoxia‐inducible factor HIF‐1 targets. ERO1 is regulated by the ER stress and HIF‐1 contributes to the invasion‐metastasis cascade in the hypoxic TME. A reduction in secretion of angiogenic factors such as VEGFA with impaired tumor angiogenesis was observed. It affected the invasion and metastasis and was mediated by a negative regulation of ATF4/CHOP.^43^

### 3.4 Tumor microenvironment (TME) modulation

During the tumor development phase, there are simultaneous changes to build a “favorable” TME. In fact, there is an active modulation of the immune response to allow the tumor to evade the immune surveillance.^38^ Moreover, transmissible ER stress (TERS), a process by which tumor cells undergoing ER stress release certain “soluble factors” inducing similar ER stress and UPR activation in surrounding cells is observed. Inducing ER stress in surrounding cells including tumor‐infiltrating immune cells modulates the TME, which can further subvert the antitumor immunity, thus promoting cancer progression and chemoresistance.^44^, ^45^ In other words, TERS by inducing ER stress and the UPR in tumor‐infiltrating immune cells impedes the development of antitumor immune response in the TME via a pro‐inflammatory response. Interestingly, the UPR genes BIP, CHOP, and XBP1s were upregulated in tumor‐infiltrating immune cells.^46^

### 3.5 Chemoresistance

The UPR arms drive chemoresistance through the five different mechanisms of acquired resistance namely: limited drug uptake, modification of the drug target, initiation of drug‐detoxifying mechanisms, mending of drug‐induced damage, and desensitization to drug‐induced apoptosis.^47^, ^48^ For example, PERK/ATF4 mediate drug efflux in hepatocellular carcinoma (HCC) via ABC cassette transporter induction.^48^ In fact, PERK was shown to have a role in sorafenib resistance through a crosstalk with lincZFAS1 signaling pathway^49^ and the same was overexpressed in resistant colon cancer cells.^50^ In addition, high IRE1α phosphorylation induced by RACK1 dysregulation inhibited sorafenib induced apoptosis in HCC.^51^ Cho et al.^52^ reported that ATF6 restricted cell death and induced chemoresistance in HCC.

In addition to the preceding, the UPR has a role in tumor quiescence and aggressiveness,^53^ ‘secretory switch’,^54^ tumor epithelial‐to‐mesenchymal transition,^54^, ^55^ tumor metabolic processes^56^ and tumor autophagy.^57^ Different components of the UPR are overexpressed in malignant cells and are responsible for chemotherapy and radiation resistance.

Several studies have reported the implication of the UPR in different tumors, such as breast cancer,^58^ atypical teratoid/rhabdoid and malignant rhabdoid tumors,^59^ Hodgkin lymphoma,^60^ pancreatic ductal adenocarcinomas,^61^ and HCC,^62^ to name a few.

Overall, the UPR is involved in different processes of tumor development. It intervenes at different phases and through different mechanisms to salvage the tumor cell from the deleterious ER stress, the harsh TME, the immune system, and promote its survival.^63^ Although many of these mechanisms need to be laid bare, the important role of the UPR in tumorigenesis and progression has been established and it has fueled several researches on its implication in different tumor development including GB.

## 4 UNFOLDED PROTEIN RESPONSE IN GLIOBLASTOMA PATHOPHYSIOLOGY

The UPR‐mediated adaptive response to ER and oxidative stress is strong in GB. The UPR sensors are upregulated in GB compared to normal brain tissue and associated with GB tumorigenesis, progression, angiogenesis, malignant phenotype, extracellular matrix remodeling, survival, and chemoresistance.^64^, ^65^, ^66^, ^67^ Moreover, the UPR is implicated in GSCs biology, thought to be the cornerstone of GB tumorigenesis and resistance. Gundamaraju et al.^68^ have reported the modulatory role of the UPR for several genes related to progression, aggressiveness, and survival in GB.

### 4.1 Angiogenesis, invasiveness and TME modulation in GB

IRE1 and PERK/ATF4 showed pivotal for the activation of VEGFA and other pro‐angiogenic genes in response to hypoxia and hypoglycemia relative to tumor ischemia.^39^ Consistent with this is the knockdown of IRE1 which inhibited VEGFA expression in GB cell lines resulting in less aggressive and smaller tumors.^40^ Furthermore, IRE1 knockdown induced the upregulation of some negative mesenchymal and invasiveness‐related modulators such as SPARC. The preceding led to reduced tumor growth, invasiveness and decreased angiogenesis, and blood perfusion with increased overall survival in xenograph mice.^69^ White et al.^70^ found that IRE1 was among the highest hypoxia DEGs in the T98G cell line and had a key role in the neovascularization and invasiveness of GB. The modulation of TME is essential for GB progression.^71^, ^72^ Khoonkari et al.^73^ reported that PERK was implicated in the extracellular matrix stiffening, essential for GB progression and adaptation to the harsh TME, through the PERK/FLNA/F‐Actin mechanism. PERK was shown to modulate the stiffness adaptation of GSCs to the harsh tumor microenvironment. In fact, high levels of PERK favored good adaptation to the TME seen by an elongated morphology, motility, and increased proliferation.^73^

### 4.2 Autophagy

The UPR arms are modulators of autophagy in GB.^74^ In fact, upon Loperamide administration, ATF4 was upregulated and mediated autophagy and autophagic cell death in GB cells.^75^

### 4.3 Stemness

UPR sensors are involved in GSC stemness maintenance,^76^ differentiation, and SOX2 level regulation^77^ and are associated with tumorigenic potential^76^ and reduced survival.^65^ The SOX2 gene regulates stemness maintenance^77^ (Figure 1). Penaranda‐Fajardo et al. found that PERK can downregulate SOX2 directly without the activation of the PERK‐UPR pathway in GSCs. The inhibition of PERK induced differentiation of GB neurospheres with persistent SOX2 levels and decreased cell adherence.^77^ On the other hand, the IRE1‐XBP1 pathway maintains GB cells' differentiated status. Accordingly, a low IRE1 activity gene signature was associated with a high level of stem cell biomarkers and vice versa in GB TCGA data sets.^78^, ^79^

### 4.4 Malignant profile and survival

The UPR downstream pathways have a role in the viability and malignant profile of GB cells. Consistent with this is the study on FK506‐binding protein 9 (FKBP9), which is overexpressed in GB and related to poor prognosis. Its silencing inhibited tumor growth in vivo and suppressed the malignant phenotype in vitro via the IRE1α‐XBP1 pathway.^80^ In addition, MTHFD2 is upregulated in glioblastoma and linked to the pathogenesis of several cancers. Its inhibition in GSCs, cell lines, and patients derived‐xenograph induced apoptosis via PERK/eIF2α pathway, whereas PERK inhibition savaged the cells.^81^ PERK was also associated with GB growth, progression, and survival in glucose‐deprived environments,^41^ and the upregulation of ATF4 in GB was linked to poor overall survival.^77^

### 4.5 Resistance

A strong UPR adaptative response to chemotherapy has been identified in GB. In fact, the knockdown of IRE1 has been associated with enhanced toxicity of celecoxib derivates,^82^ arsenic trioxide, hydrogen peroxide, and ROS inducers.^83^ Moreover, PERK silencing sensitized glioma cells to apoptosis induction in hyperglycemic conditions. Following irradiation, ATF6 silencing attenuated clonogenic survival and increased apoptosis.^67^ A resistant phenotype of GB to the sarcoendoplasmic reticulum Ca2+ ATPase inhibitor 12ADT was strongly associated with the expression of IRE1 and ATF4.^8^ GRP78 overexpression has been reported to increase GB cell resistance, while its silencing sensitized the same to chemotherapies and radiation.^84^, ^85^

Overall, the previous discussion informs on the different roles of the UPR arms in the development, survival, and recurrence of GB. In addition, it is associated with GB therapeutic resistance, suggesting it as potential target for treatment as discussed below.

## 5 THE UNFOLDED PROTEIN RESPONSE IN GB TREATMENT RESISTANCE AND AS DRUGGABLE TARGET

The resistance to treatment can be inherent or acquired. During the course of the treatment, some tumor cells acquire resistant patterns to overcome the additional stress of the drug and thrive. Factors and mechanisms such as the activation of pro‐survival pathways, enhanced drug efflux, alteration in the drug target, TME modulation among others are responsible for the adaptation of tumor cells to chemotherapeutic agents induced‐stress.^86^ The adaptative response induced by the UPR arms in response to treatment is reported to be very strong in GB. In fact, the UPR sensors are linked to CSCs sphere‐forming ability post‐chemotherapy,^53^ increased cell survival during ER stress,^87^ resistance to treatment, and modulation of the sensitization to chemotherapeutic agents including TMZ.^88^

**FIGURE 2:**
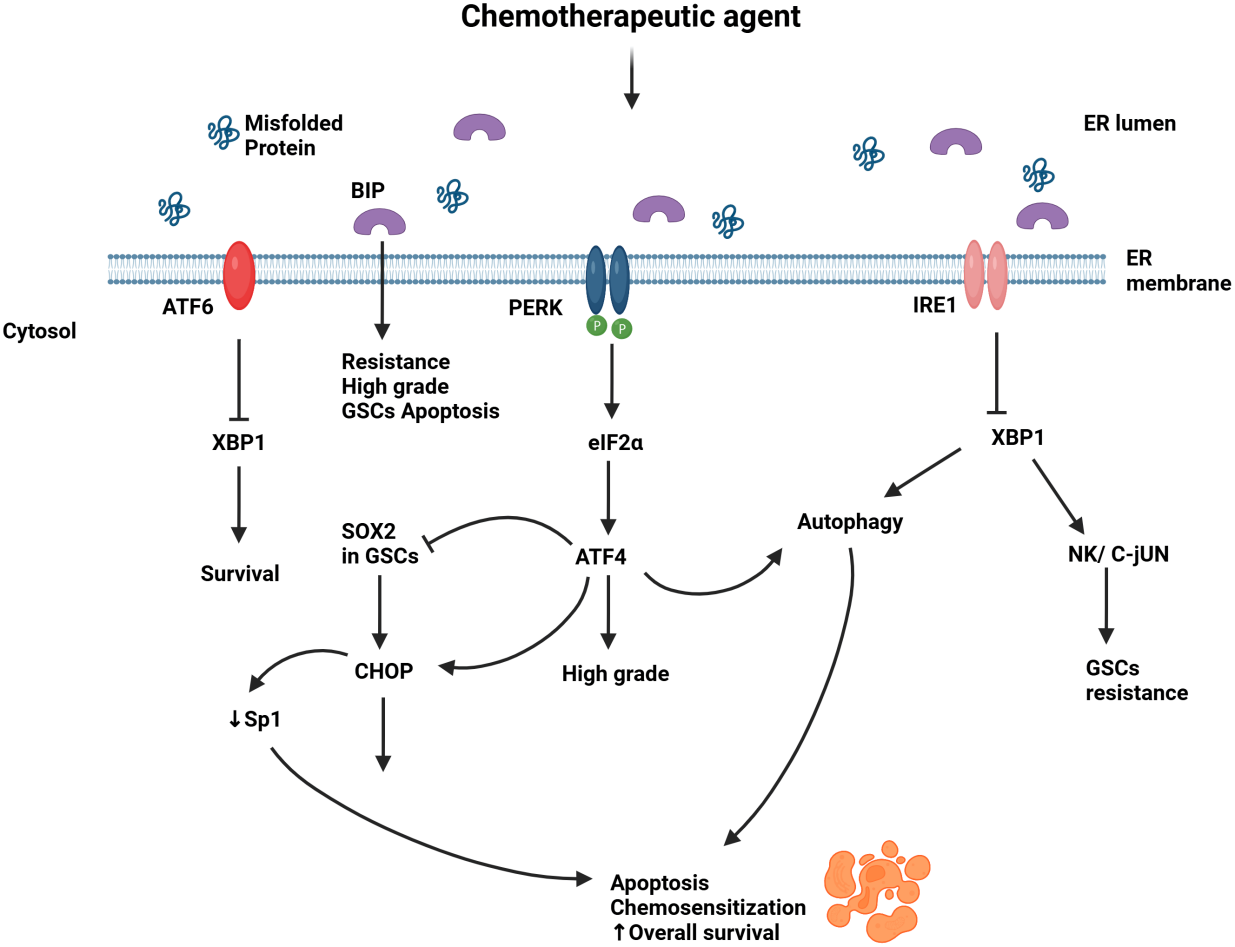
The unfolded protein response in GBM treatment resistance. Upon administration of oncolytic treatment, IRE1 and PERK are phosphorylated. IRE1 via NK/c‐Jun induces resistance in GSCs and apoptosis. PERK can induce apoptosis either directly via CHOP or through downregulation of SOX2 in GSCs. ATF6 promote survival. GRP78 is linked to resistance, aggressiveness and apoptosis. Created with BioRender.com.

Given that GB cells can get through different types of stresses namely metabolic and therapeutic by taking advantage of the UPR, its arms have been studied and targeted to overcome the treatment resistance (Figure 2).

### 5.1 UPR in chemoresistance and as a target

*IRE1/XBP1* arm is known for promoting a pro‐survival and drug resistant effect in GB cells. Consistent with this is the study on the inhibition of IRE1 using the IRE1 RNase inhibitor MKC886 combined with irradiation/chemotherapy which resulted in an extension of the overall survival in vivo with the sensitization of GB cells to irradiation/chemotherapy.^89^ Moreover, IRE1 deletion by CRISPR increased the responsiveness of the human GB cell line U251 to 12ADT, a synthetic analog of thapsigargin, an inducer of ER stress.^8^ GSCs are pivotal for GB chemoresistance and recurrence.^90^ Booth et al.^82^ found that the knockdown of IRE1 or XBP1 enhanced the cytotoxic effect of OSU‐03012 alone or with sildenafil in GSCs and GB cells via the caspase‐dependent pathway. On the other hand, high XBP1 was associated with reduced survival.^65^ Furthermore, the upregulation of GRP78 and IRE1α phosphorylation was proportional to the intensity of GB resistance. Hence, TMZ‐resistant cells tolerated the ER stress induced by Thapsigargin.^64^ The very recent discovery of the FDA‐approved drugs cefoperazone, methotrexate, folinic acid, and fludarabine phosphate as IRE1 inhibitors has shown effective in sensitizing human cell models of GB to TMZ.^91^ In addition, IRE1 inhibitor Z4P successfully penetrated the blood–brain barrier, impaired GB growth, and prevented the recurrence in vivo when administered as an adjuvant of TMZ.^92^

*PERK* levels are associated with tumor grade.^41^ Betulinic acid (BA) increased PERK/CHOP signaling with a reduction in the transcription factor specificity protein 1 (Sp1) expression. TMZ‐resistant cells exhibited elevated Sp1 which induced PERK/CHOP pathway inhibition and protected the GB cells against the chemotherapeutic agent.^93^ In other words, PERK/CHOP overexpression by BA decreased Sp1 in GB resistant cells and induced apoptosis. Additionally, Dastghaib et al.^88^ have shown that PERK and IRE1 signaling pathways modulated Simvastatin sensitization of GB cells to TMZ‐induced apoptosis.

*GRP78* overexpression is linked to high‐grade glioma, GB cells protection from stress, and increased resistance to some chemotherapeutic agents.^84^, ^85^ On the other hand, the silencing of GRP78 sensitized glioma cells to the same agents.^84^ Interestingly, TMZ can induce GRP78 overexpression.^85^ This was exploited in a recent study combining TMZ and targeted suicide gene therapy by the RGD4C/AAVP‐GRP78. The systemic administration of RGD4C/AAVP‐GRP78 in mice targeted intracranial tumors and the addition of TMZ exerted a synergic apoptotic effect on orthotopic GB.^94^ Moreover, targeting *GRP78* with antibodies has shown promising. In fact, Dadey et al. observed a reduced cell proliferation, colony formation with increased apoptosis in GB cells consecutive to GRP78 downregulation with antibodies. Reducing GRP78 expression suppressed PI3K/Akt/mTOR signaling, responsible for radiation resistance. Thus, the administration of ionizing radiation in addition to anti‐GRP78 antibodies resulted in delayed tumor growth in GB heterotopic tumor models.^95^

*SEL1L* is an UPR‐associated degradation protein that modulates cell proliferation, cell fate decision, ER homeostasis, neural stem cell maintenance, and differentiation. Cattaneo et al. found that SEL1L knockdown sensitized GSCs to the apoptotic effects of valproic acid (VA) with decreased GSCs proliferation and neurosphere size and enhanced differentiation. In addition, exacerbated ER stress and further UPR activation with GRP78 and elevated CHOP were observed in some GB cell lines upon VA administration.^96^

### 5.2 The UPR mediating apoptosis

Several studies have reported the UPR as a mediator in the apoptotic mechanism of some chemotherapeutic agents. For example, melitherapy with 2‐hydroxycervonic acid (HCA) has proven effective on GB cells and xenograft models. The overexpression of the UPR arms and induction of BiP and CHOP were observed. HCA‐induced autophagy and its action were mediated by the JNK/c‐Jun/CHOP pathway.^97^ Moreover, CAY10566 (CAY) administered intranasally, in GSCs xenograph mouse models, led to a toxic accumulation of fatty acids. Subsequent decreases in tumor proliferation, cell death, and extended survival in xenograph mice were observed. CAY action was mediated by an increase in GRP78, sXBP1, GADD34, and CBaHOP, and the phosphorylation of eIF2a and g‐H2AX was noted. Additionally, the combination of CAY and TMZ showed a synergic cytotoxic effect (103). The same was reported for drugs such as NCL‐1 and NCD‐38 survival,^98^ methylenetetrahydrofolate dehydrogenase/cyclohydrolase (MTHFD2) (73), TAK‐243 (80) to name a few. The administration of tunicamycin increased CHOP with reduced self‐renewal and colony formation partly through SOX2 downregulation in GSCs.^76^ Furthermore, GSCs expressed increased ER stress markers GRP78, CHOP, and EIF‐2a within 24 h of treatment with shikonin compared to the control group. In addition, 4‐phenylbutyric acid, an ER stress inhibitor decreased GRP78 levels, reduced cell viability, and potentiated shikonin cytotoxic effects in vivo resulting in smaller tumors and increased viability in GSCs‐xenograft mice.^99^

### 5.3 UPR mediating radiation‐induced apoptosis

The UPR has a role in cancer cells responses to radiation.^100^, ^101^ Poorly characterized GSCs escape the treatment‐induced stress and cause the frequent relapse and chemo‐radio resistance seen in GB.^90^ Shah et al. reported that radioresistance in GSCs was achieved by the overexpression of ER stress and overcome through the overactivation of UPR. On this basis, pharmacological modulation of the UPR or generation of ER stress aiming at radiosensitizing GB cells has shown good results. For example, celecoxib combined with γ‐irradiation increased autophagy in GB cells via ER stress,^102^ and GSK2606414 successfully inhibited PERK and subsequently reduced up to 80% of the radiation‐induced downstream genes.^103^ Four clinical trials on the effectiveness of Celecoxib in combination with TMZ or radiation have been conducted (ClinicalTrial.gov).

### 5.4 UPR and drug repurposing in recurrent GB

Disulfiram (DSF), initially approved as an anti‐alcoholism drug, combined with copper, has proven effective in augmenting the therapeutic effect of TMZ.^104^ In fact, DSF/Cu‐induced autophagy‐dependent apoptosis via the activation of the UPR.^105^ The overall survival in GSC models derived from patients with newly diagnosed or recurrent tumors was markedly increased after DSF use.^104^ There are several clinical trials on the effectiveness of DSF for GB (ClinicalTrials.gov).

Collectively, these studies have shown the effectiveness of targeting the UPR against GB in vivo and in vitro and its implications in the mechanism of action of chemotherapeutic agents with some having conducted clinical trials (Table 1). However, more studies are needed fully to understand how specific arms of the UPR are activated in response to specific endogenous and exogenous stressors has to be fully understood in order to have a targeted and expected response. Furthermore, there is a need to identify the most effective chemotherapeutic agents based on their mechanisms of action, converge the focus on them through in vitro and in vivo studies with the hope to reach a translational step.

**TABLE 1:** Antimitotic agents targeting the UPR for GB treatment.

Cytotoxic agents	Mechanism	References
NCL‐1 and NCD‐38, KDM1A inhibitors	Activation of the UPR	98
CAY10566 (CAY)	Exacerbation of ER stress through the accumulation of SFAs	106
Cefoperazone, methotrexate, folinic acid, and fludarabine phosphate	Impact on IRE1 activity	91
Betulinic acid (BA)	Sp1‐mediated PERK/CHOP signaling inhibition	93
2‐Hydroxycervonic acid (HCA)	Activation of the JNK/c‐Jun/CHOP/BiP axis	97
Celecoxib	ER stress loading on GB cells	102
2‐deoxy‐D‐glucose (2‐DG)	Overactivation of ER stress‐related pathways	107
CCF642	Protein disulfide isomerase via downregulation of PERK signaling	108
Cudraflavone B	PERK/ATF4/CHOP pathway	109
Remdesivir	PERK‐mediated UPR	110
ABTL0812	Activation of ER stress UPR	111
Tetralol derivative NNC‐55‐0396	Activation of IRE1α resulting in the nuclear translocation of CHOP	112
Chlorpromazine	ER and UPR activation	113
TAK‐243, UBA1 inhibitor	Global protein ubiquitination disruption with PERK/ATF4 and IRE1α/XBP activation	114
Simvastatin	Increase in GRP78, XBP splicing, eIF2α phosphorylation, and inhibition of autophagic flux	88
Gamitrinib and romidepsin	Stress‐response activation	115
12ADT	CRISPR‐mediated deletion of the ERN1, IGFBP3, IGFBP5 signature genes	8
Phenylbutyrate	Upregulation of CHOP and downregulation of BIP	116
PES‐Au@PDA: HSPA5 inhibitor (pifithrin‐μ, PES) and radiosensitizer (gold nanosphere, AuNS)	Activation of proapoptotic UPR cascades	117
Withaferin A	ER stress induced through the ATF4‐ATF3‐CHOP axis	118
5‐LO inhibitor derivative 3‐tridecyl‐4,5‐dimethoxybenzene‐1,2‐diol hydroquinone (EA‐100C red)	CHOP and Beclin1 upregulation and activation of caspases 3, 9, JNK and NF‐kappa B pathway	119
Curcumin inspired bis‐chalcones	Induction of ER and UPR	120
PDI inhibitor pyrimidotriazinedione 35G8	Nuclear factor‐like 2 (Nrf2) antioxidant response, endoplasmic reticulum (ER) stress response, and autophagy	121
Endothelial‐monocyte activating polypeptide II (EMAP II)	Upregulation of GRP78, eIF2α, and CHOP	122
NIM811, a small‐molecule cyclophilin‐binding inhibitor	Paraptosis due to unresolved ER stress	123
Radicol (RAD)	Attenuation of protein disulfide isomerase (PDI) and induction of the UPR and lethal ER stress	124
Tubastatin A (TUB, a selective inhibitor of HDAC6)	Proapoptotic signals of the UPR and ER stress	64
Curcumin analog C‐150	NF‐κB, UPR, and Akt/Notch Pathways	98
High linear energy transfers	Mediation of autophagy via the UPR‐eIF2α‐CHOP‐Akt signaling axis	125
Honokiol (HNK)	Binding to unfolded conformations of the GRP78	126
Fusion protein EGF‐SubA	Selective cleavage of GRP78	127
Dual tumor‐targeted phage containing the arginine‐glycine‐aspartic acid tumor homing ligand and GRP78 promoter	UPR signaling cascade	128
Antp‐TPR hybrid peptide	Increase UPR	129
albumin‐encapsulated nanoparticles	Downregulation of PERK	108
Z4P	Inhibition of IRE1	92

*Note*: This table summarizes the different studies on components targeting the UPR with an overview of the respective mechanisms.

Abbreviations: ATF6, activating transcription factor 6; BIP, GRP78; CHOP, C/EBP homologous protein; ERNI, IRE1; GRP78, 78 kDa glucose‐regulated protein; IRE1, inositol‐requiring enzyme 1; KDM1A, lysine‐specific demethylase‐1; NCD‐38, (2‐(N‐4‐phenylbenzenecarbonyl)amino‐6‐(trans‐2‐phenyl‐cyclopropane‐1‐amino‐N‐(3‐chlorobenzyl)hexaneamide trifluoroacetate)); NCL‐1, (N‐[(1S)‐3‐[3‐(trans‐2‐aminocyclo‐propyl)phenoxy]‐1‐(benzylcarbamoyl)propyl] benzamide); PERK, protein kinase RNA‐like ER kinase; SFAs, saturated fatty acids; UBA1, ubiquitin‐activating enzyme 1; UPR, unfolded protein response.

## 6 CONCLUSION

The UPR is integral to GB genesis and development. It governs GB cell homeostasis and helps the cell to overcome ER stress, cope with several stressors, and thrive in hostile environments such as hypoglycemic and hypoxic environments. IRE1, PERK, and GRP78 are associated with tumor grade and resistance to treatment. Targeting the UPR arms has shown promising in inducing apoptosis, overcoming resistance in GB, and has been found to mediate apoptosis for several cancer drugs. Thus, chemotherapeutic agents with the ability to modulate the UPR are potential GB treatment components. However, the balance between the ER stress regulator and apoptosis effector function, and the extent to which the UPR needs to be persistently overexpressed to switch to its apoptotic function need to be clearly understood. Moreover, the UPR branches can have parallel effects in response to a stressor. Thus, the specificity of the target agent should be high enough to avoid cross‐effects from the other branches. In addition, one will have to navigate the crosstalk between the physiological processes in regard to UPR functions in order to avoid systemic adverse effects. Hence, more preclinical and translational studies are needed to confirm the safety and effectivity of the potential targeted therapies and their ideal delivery route to minimize systemic toxicity while optimizing target coverage and specific local tumor effects. The development of more specific targets and stratification of patients with the identification of a subset more likely to benefit from the intervention will open avenues for effective clinical translation.

## AUTHOR CONTRIBUTIONS

Lucette Z. Simbilyabo and Liting Yang designed the review, Lucette Z. Simbilyabo, Liting Yang, and Jie Wen wrote the paper, Lucette Z. Simbilyabo and Jie Wen drew the figures, Lucette Z. Simbilyabo designed and drafted the table, Zhixiong Liu and Liting Yang proofread the paper, all the authors approved the final version of the manuscript.

## CONFLICT OF INTEREST STATEMENT

The authors declare no conflicts of interest.

## Data Availability

Data sharing is not applicable to this article as no datasets were generated or analyzed during the current study.
